# Feeding Difficulties and Feeding Behaviors of Thai Children with Cow's Milk Protein Allergy

**DOI:** 10.1155/2023/6630167

**Published:** 2023-11-22

**Authors:** Kununya Charoensriwattanakul, Kamolmart Wannaphahoon, Sirinuch Chomtho, Pantipa Chatchatee, Narissara Suratannon, Pannipa Kittipongpattana, Orapa Suteerojntrakool

**Affiliations:** ^1^Department of Pediatrics, Faculty of Medicine, Chulalongkorn University and King Chulalongkorn Memorial Hospital, Bangkok 10330, Thailand; ^2^Pediatric Nutrition Research Unit, Division of Nutrition, Department of Pediatrics, Faculty of Medicine, Chulalongkorn University, Bangkok 10330, Thailand; ^3^HAUS IAQ Research Unit, Division of Allergy, Immunology and Rheumatology, Department of Pediatrics, Faculty of Medicine, Chulalongkorn University, King Chulalongkorn Memorial Hospital, the Thai Red Cross Society, Bangkok 10330, Thailand; ^4^Center of Excellence for Allergy and Clinical Immunology, Division of Allergy, Immunology and Rheumatology, Department of Pediatrics, Faculty of Medicine, Chulalongkorn University, Bangkok 10330, Thailand; ^5^Division of Ambulatory Pediatrics, Department of Pediatrics, Faculty of Medicine, Chulalongkorn University, Bangkok 10330, Thailand

## Abstract

**Background:**

Cow's milk protein allergy (CMPA) is a common food allergy in infants and young children and may be a risk factor for feeding difficulties. Studies exploring feeding difficulties and feeding behaviors in Thai children with CMPA are scarce.

**Objectives:**

To determine the prevalence of feeding difficulties and feeding behaviors in Thai children with CMPA compared to healthy controls.

**Methods:**

A cross-sectional study was performed comparing children aged 1-6 years old diagnosed with CMPA and had eliminated cow's milk for at least 4 months with age-matched healthy children. Feeding difficulties were evaluated using the Montreal Children's Hospital Feeding Scale questionnaire, and feeding behaviors were measured using the Child Eating Behavior Questionnaire (CEBQ).

**Results:**

One hundred and twenty-two participants were recruited (30 children with CMPA and 92 controls). The median age of the CMPA and control groups was 31.0 (14.0, 43.3) and 40.0 (28.0, 53.8) months, respectively (*p* value = 0.01). The CMPA group had lower calcium, phosphorus, and zinc intake than the healthy controls. The prevalence of feeding difficulties between the two groups did not show a significant difference (36.7 vs. 43.5%, *p* value = 0.70). The slowness in the eating subscale of feeding behaviors exhibited a lower score in the CMPA group than in the healthy group. Feeding difficulties was positively correlated with the desire to drink (*β* 3.079, *p* value = 0.011) but negatively correlated with the enjoyment of food subscale of CEBQ among the CMPA children (*β* -10.684, *p* value < 0.001).

**Conclusion:**

Despite a similar prevalence of feeding difficulties between CMPA and healthy children, the CMPA group demonstrated some differences in feeding behaviors. The lower score of enjoyment of food and a higher score of desire to drink correlated with a higher degree of feeding difficulties in the CMPA children. The provision of appropriate nutrition for this group of children should be prioritized.

## 1. Introduction

The term feeding difficulties can be broadly defined as a variety of feeding problems such as prolonged mealtimes, crying/irritability at feeding time, food refusal, throwing and pushing away food, disruptive and stressful mealtimes, a lack of appropriate independent feeding, the introduction of distractions to increase intake, or failure to introduce advanced textures [1, 2]. These problems are usually found in infants and young children. The estimated prevalence of feeding difficulties in healthy Thai children has been reported as 28.1% at the age of two, with a gradual decrease at three (26.9%) and four (19%) [3]. Children with feeding difficulties are at risk of poor growth, nutritional deficiency, cognitive impairment, and emotional dysfunction [4].

The common causes of feeding difficulties can be divided into two main factors: child and caregiver factors. Child factors leading to feeding problems include birth weight, organic causes, and traumatic feeding [5]. Previous literature found a high prevalence of feeding difficulties among preterm infants or infants who are small for their gestational age. Parents may perceive these infants as too small and in need of extra calories to grow, leading to overfeeding beyond their hunger cues. Organic causes such as gastroesophageal reflux disease, vomiting, and swallowing problems may also affect feeding. Mathisen et al. [6] found that gastroesophageal reflux infants have moderate to severe oral motor function, significantly less self-feeding, and more feeding difficulties than normal infants. Traumatic feedings, including choking or a history of receiving tube feeding, can increase the risk of feeding difficulties. Apart from the child factors, caregiver factors such as transitional feeding (transition from one method to another) and mechanistic feeding (precise timing of food without waiting for hunger cues) can also prompt feeding difficulties [5].

Cow's milk protein allergy (CMPA) is a common food allergy found in infants and young children. Previous studies reported that the estimated global prevalence of CMPA is 5.2% [7], with a prevalence of CMPA in Thailand of 1.7% [8]. Extensive research has shown that the prevalence of food allergy peaks in early childhood (1-2 years of age) and declines afterwards. The symptoms and signs of CMPA are varied in each child. The most frequently reported symptoms were gastrointestinal in nature (51%) (nausea, vomiting, and diarrhea), followed by skin rashes and urticaria (35%), angioedema (12%), and respiratory symptoms (2%) [8].

Data from previous studies suggest that children with CMPA may be at risk of feeding difficulties. It is possible, therefore, that repeated painful experiences, such as reflux, vomiting, and constipation, can result in dysphoric feeding times and feeding difficulties. Furthermore, the delayed diagnosis of food allergies, especially in a critical period of oral motor skill development during the 7-18-month period, may lead to the delayed introduction of new tastes and textures. The bitter taste of hypoallergenic formula, a mainstream treatment of CMPA, can affect dietary avoidance and limit the variety of foods [9]. Most previous studies exploring the feeding difficulties in children with CMPA were performed in Western countries, where cow's milk and dairy products are staple foods. The culinary culture and feeding practices of caregivers between Asian and Western countries are significantly different and warrant study among Asian populations.

To the best of our knowledge, there are no studies regarding feeding difficulties and feeding behaviors in Thai children with CMPA. In general, only a few previous studies investigated feeding behaviors using validated questionnaires and explored the risk factors for feeding difficulties in CMPA children. This study is aimed at determining the prevalence of feeding difficulties and exploring the feeding behaviors of Thai children with CMPA compared to those in the healthy group. Our study also assessed factors associated with feeding difficulties in Thai children with CMPA.

## 2. Methods

### 2.1. Study Design and Participants

This cross-sectional study was conducted between October 2021 and November 2022 at King Chulalongkorn Memorial Hospital, Bangkok, Thailand. The participants were recruited from allergy clinics, general outpatient clinics at King Chulalongkorn Memorial Hospital, and social networks.

The inclusion criteria were 1- to 6-year-old Thai children born full term (gestational ages 37 to 41 + 6 weeks) with a 2500 to 4000 gram birth weight. Children with any chronic diseases such as neurologic, cardiac, pulmonary, liver, renal, hematologic, oncologic, genetic, metabolic, and autoimmune diseases were excluded from this study. The subjects were divided into two groups: a CMPA group and a healthy control group. The CMPA group included any child who had received a diagnosis of cow's milk protein allergy (CMPA) through either a regimen of cow's milk elimination followed by a rechallenge procedure or via the detection of a positive specific IgE or skin prick test, accompanied by the manifestation of clinically suspected symptoms upon cow's milk ingestion as assessed by pediatric allergists. In addition, all children in the CMPA group had to eliminate cow's milk and dairy products from their diet for at least 4 months to be eligible for the study. The control children were healthy children on the date of enrollment. Written informed consent was obtained from all participants' parents.

This study was approved by the Institutional Review Board of the Faculty of Medicine, Chulalongkorn University (IRB no. 233/64, COA no. 1040/2021).

### 2.2. Demographic Data, Dietary Intake, and Anthropometry

Demographic data, including age, sex, perinatal history (gestational age, birth weight), duration of receiving exclusive breastfeeding, age of starting complementary food, and caregiver's information (main caregiver, educational level, and family income), were collected by the researchers in a face-to-face interview. Data regarding the details of CMPA such as the child's age at diagnosis, age at the elimination of cow's milk and dairy products, number of food allergies, and allergic symptoms were also collected for the CMPA group.

Dietary intake was evaluated by a well-trained dietician using a 24-hour dietary recall. The dietary data were computed to explore macronutrient and micronutrient intake using INMUCAL-Nutrients V. 4.0 software developed by the Institute of Nutrition, Mahidol University, Thailand.

Anthropometric data (weight, length for the children <two years old, and height for the children aged ≥two years old) were measured using a digital weighing scale (Seca GmbH & Co. KG, Seca 703, Germany). Body mass index (BMI) was calculated using weight divided by height or length squared. All measurements were performed twice, and averages were used for analysis. The anthropometric data were analyzed using World Health Organization (WHO) Anthro software (WHO Anthro v.3.2.2 for children aged 12 to 60 months and WHO Anthro Plus v.1.0.4 for children aged 61 to 83 months) to convert to age- and sex-specific *z*-scores.

### 2.3. Feeding Difficulties and Feeding Behaviors

Feeding difficulties and behaviors were determined using self-administered questionnaires completed by the parents. Feeding difficulties were evaluated by the Thai version of the Montreal Children's Hospital Feeding Scale (MCH-FS) questionnaire, which was translated into Thai language and validated by Benjasuwantep et al. [10]. MCH-FS consisted of 14 questions with topics that included feeding (oral motor, oral sensory, and appetite), mealtime behaviors, maternal concerns about feeding, maternal strategies employed to address feeding problems, and family reactions [10]. Answers to questions were tabulated and converted into *T*-scores and then divided into 4 groups: (1) no difficulties: *T*-score < 61; (2) mild difficulties: *T*-score 61-65; (3) moderate difficulties: *T*-score 66-70; and (4) severe difficulties: *T*-score > 70.

Feeding behaviors were measured using the validated Thai version of the Child Eating Behavior Questionnaire (CEBQ), with a Kaiser-Meyer-Olkin value of 0.865 and Cronbach's alpha ranging from 0.64 to 0.80 [11]. The CEBQ is comprised of 35 questions that measure the frequency of children's behaviors and experiences on a 5-point scale: 1 = never, 2 = rarely, 3 = sometimes, 4 = often, and 5 = always. The feeding behaviors were assessed on 8 subscales: food responsiveness, emotional over-eating, enjoyment of food, desire to drink, satiety responsiveness, slowness in eating, emotional under-eating, and food fussiness. The average scores of each question in each subscale were computed and analyzed.

### 2.4. Statistical Analysis

Statistical analysis was performed using IBM SPSS statistics version 22.0 (IBM Corp., Armonk, NY, USA). The normality of the data was checked using the Kolmogorov-Smirnov test before analysis. Data with a normal distribution included birth weight, weight for age *z*-score, height for age *z*-score, BMI for age *z*-score, phosphorus intake, and MCH-FS *T*-score. Categorical data including gender, caregiver's information (main caregiver, educational level, and family income), number of food allergies, allergic symptoms, and MCH-FS interpretation were shown as number and percentage, while continuous data including age, gestational age, birth weight, duration of receiving exclusive breastfeeding, age starting complementary food, anthropometry data, age at diagnosed CMPA, age at the elimination of cow's milk and dairy products, and dietary data were expressed as mean ± SD or median (IQR 1, 3), as appropriate. The comparison of data between the CMPA and control groups was analyzed using the independent *t*-test, the Mann–Whitney *U*-test, or the chi-squared test. MCH-FS *T*-scores were adjusted for age. A univariate linear regression analysis was performed to determine factors associated with feeding difficulties. Factors with a *p* value from the univariate regression <0.10 were retained in the multiple regression by the backward elimination procedure. A *p* value of less than 0.05 was considered to be statistically significant.

## 3. Results

One hundred and fifty-nine children were recruited, but thirty-seven children were excluded from the study because 4 children did not meet the inclusion criteria and 33 were denied participation due to their inconvenience in answering the questionnaire. A total of 122 children were enrolled for the analysis.

### 3.1. Demographic Data

Baseline characteristics and anthropometric data are shown in Table 1. Participants in the CMPA group were significantly younger than those in the healthy group (31.0 (14.0, 43.3) vs. 40.0 (28.0, 53.8) months old, *p* value = 0.01). There were no differences between the two groups in gestational age, birth weight, sex, duration of exclusive breastfeeding, age of starting complementary food, main caregivers and their educational level, and family income. No differences in weight for age, height, or length for age and BMI for age *z*-score between the CMPA and healthy groups were found (*p* value > 0.05).

Table 2 shows the characteristics of the children with CMPA. The average age at diagnosis of CMPA was 7.0 (3.8, 10.5) months, and the average age of starting cow's milk elimination was 6.0 (3.0, 9.0) months. Twenty-seven individuals were identified as having immunoglobulin E (IgE)-related allergies, while 3 individuals exhibited a non-IgE nature. Around two-thirds of the CMPA children had multiple food allergies. During cow's milk elimination, nearly half of the children received more than one type of milk (such as mixing soy milk and formula), and two-fifths of the children received an extensively hydrolyzed or amino acid formula. Half of the children presented with gastrointestinal symptoms.

### 3.2. Dietary Intake

Participants in the CMPA group had a lower calcium, phosphorus, and zinc intake and a higher iron intake than the healthy group (*p* value < 0.05) (Table 3). However, the two groups had no significant differences in total calories and protein intake (*p* value 0.13 and 0.34, respectively).

**Figure 1:**
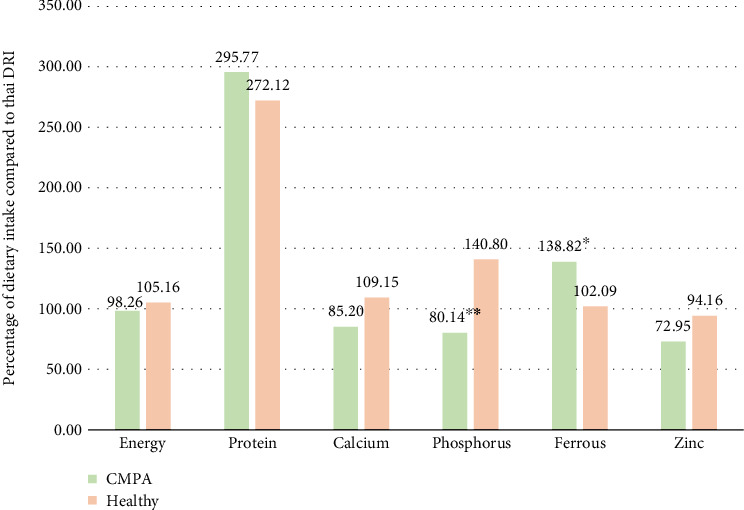
Percentage of dietary intake according to Thai Dietary Reference Intake (DRI) comparing the CMPA and healthy group. The comparison between the CMPA and healthy group was performed by an independent *t*-test. ^∗^*p* value < 0.05, ^∗∗^*p* value < 0.001. Abbreviation: CMPA, cow's milk protein allergy; DRI, dietary reference intake.

Details of the dietary intake of both groups compared to the Thai Dietary Reference Intake (DRI) are shown in Figure 1. Sufficient calcium and phosphorus intake in the CMPA group was not achieved according to the Thai DRI and was lower than in the control group. Zinc intake in both groups was lower than the requirement. Additionally, the CMPA group had a significantly lower phosphorus intake and a higher iron intake compared to the healthy group (*p* value < 0.05).

### 3.3. Feeding Difficulties and Feeding Behaviors

**Figure 2:**
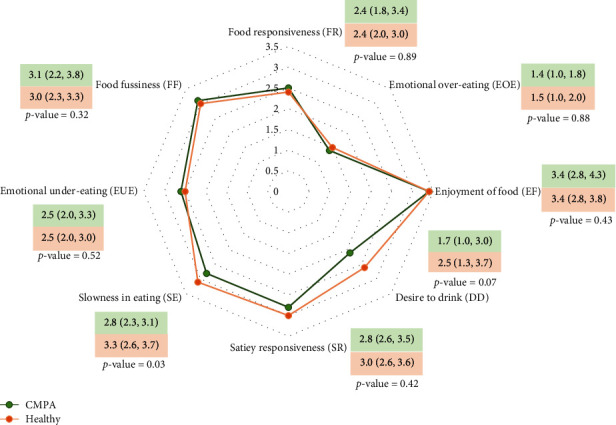
Feeding behaviors of the children with CMPA compared to the healthy group. The comparison between the two groups by using the Mann–Whitney *U* test.

Table 4 shows the comparison of feeding difficulties and feeding behaviors between the children with CMPA and healthy controls. There was no statistical difference in feeding difficulties *T*-scores between the two groups. The prevalence of feeding difficulties was 36.7% in the CMPA group, while the healthy group was 43.5%, but no significant difference was found (*p* value = 0.70). Feeding behaviors assessed by CEBQ showed that the CMPA group had a lower score in the slowness in the eating subscale of feeding behaviors compared to the healthy group (*p* value = 0.03). No notable differences were observed in the other subscales (Figure 2).

### 3.4. Factors Associated with Feeding Difficulties

Factors associated with higher *T*-scores of MCH-FS are shown in Tables 5 and 6. The higher feeding difficulties scores were significantly correlated with all CEBQ feeding behaviors subscales, except for emotional over-eating and emotional under-eating. In the multiple linear regression, only the slowness in eating and food fussiness subscales remained significantly and positively correlated with the feeding difficulties *T*-score. A negative correlation was found between the enjoyment of food subscale and the feeding difficulties *T*-score.

Regarding the factors associated with a higher feeding difficulties score in the CMPA group (Table 6), all CEBQ feeding behaviors subscales except for emotional under-eating were found to be significantly associated with feeding difficulties in the univariate regression. After multiple linear regression was applied, a positive correlation was found only between the desire to drink subscale and feeding difficulties, while the enjoyment of food subscale had a statistically significant and negative correlation with the feeding difficulties *T*-score.

## 4. Discussion

This is the first study in Thailand to compare the prevalence of feeding difficulties in Thai children with CMPA to a healthy group of children using validated questionnaires. The results showed no significant differences in the prevalence of feeding difficulties between the CMPA group and controls. The slowness in the eating subscale of feeding behaviors did show a lower score in the CMPA group compared to the healthy group. The CEBQ feeding behaviors subscales except for emotional under-eating were associated with feeding difficulties *T*-scores after univariate regression analysis. In the multiple regression model, a lower score of enjoyment of food and a higher score of desire to drink remained associated with a higher degree of feeding difficulties in the CMPA children.

Our study found no significant difference in the prevalence of feeding difficulties between the CMPA group (36.7%) and the healthy controls (43.5%). This finding is contrary to previous studies that suggested feeding difficulties among CMPA children were higher than those of healthy children. For example, a study by Maslin et al. [2] which enrolled 66 children with CMPA and 60 healthy children found that the prevalence of feeding difficulties assessed by the MCH-FS questionnaire among CMPA children was 13.6%, while the prevalence in healthy children was only 1.6%. Another study by Rodrigues et al. [12] performed on 146 children with CMPA and 109 healthy children found that the feeding difficulties score assessed by the MCH-FS questionnaire was higher in the CMPA group than in the healthy group. Nevertheless, the prevalence of feeding difficulties between the two groups was not different (32.1% and 28.4%, respectively, *p* = 0.541). A possible explanation for our results may be the small sample size of the CMPA group. Additionally, most healthy children in this study were enrolled using social media, which may recruit children whose parents have greater concern about their eating behaviors. The presence of selective bias cannot be ruled out.

Our observations revealed that the CMPA group exhibited a decreased score in the slowness in the eating subscale compared to the healthy group. No significant distinctions were identified in the remaining subscales. This result is contrary to the study of Ercan and Tel Adıgüzel [13], who reported that the food allergy group had lower scores on the emotional over-eating, slowness in eating, and food fussiness subscales, as well as higher scores on the food avoidance total score and satiety responsiveness subscales, compared to the control groups. Data presented by Maslin et al. [14] showed, however, different patterns in the slowness in eating subscales, with higher scores in the CMPA group than in the healthy group. Both previous studies were performed in Western countries where cow's milk and dairy products were the main food sources. The hypothesis of a different result from the previous studies is that cow's milk and dairy products are not staple foods in Thailand and may not affect Thai feeding behaviors. There are also due to differences in the culinary culture, types of foods, and feeding practices of caregivers between Asian and Western countries.

Our results also revealed that the lower scores on the enjoyment of food subscale and the higher scores on the slowness in eating and food fussiness subscales were significantly correlated with higher feeding difficulties scores in all participants. These results are in line with those obtained by Rogers et al. [15], who found a significant correlation between the MCH-FS scores and the maternal report of the satiety responsiveness, enjoyment of food, food responsiveness, slowness in eating, and food fussiness subscales. Our study also found that the enjoyment of food and desire to drink subscales in the CMPA group were associated with the feeding difficulties scores. This may be explained by the fact that children with CMPA may be limited in the variety of complementary foods during late infancy, which is the critical period of new food acceptance. Therefore, they may prefer liquid food such as milk or juice more than solid food, which may affect the degree of feeding difficulties [16].

Children with CMPA tend to have significantly lower calcium, phosphorus, and zinc intake but higher iron intake compared to the healthy group. Similar trends have been reported by Boaventura et al. [17] in their work on the CMPA group showing a lower intake of calcium and lipids. These results are similar to those of Ercan and Tel Adıgüzel et al. [13], who found that children with CMPA had lower calcium, folic, vitamin B1, and vitamin C intake than the control group. This can be explained due to the elimination of cow's milk and dairy products which are the main sources of calcium, phosphorus, and zinc. Our study results on iron intake are concordant with those of Grażyna et al. [18], who found that the CMPA group had a higher iron intake than the healthy group. This might be because most of the children with CMPA received the hydrolyzed formula, which is fortified with iron [16, 18].

This is the first study in Thailand to assess feeding difficulties and feeding behaviors in children with CMPA compared to healthy children. An additional strength of the study is the use of validated questionnaires to explore the prevalence of feeding difficulties and feeding behaviors. There are some limitations. Firstly, the healthy group was older than the CMPA group, which may have affected the results. However, age was controlled before comparing the feeding difficulties scores between the two groups. Furthermore, it is important to note that this study was performed at a single center, and the majority of the participants were from Bangkok and its vicinity, which may limit its generalizability. Nevertheless, the geographical variability may not necessarily impact feeding behaviors. Social media was also employed to enroll most of the children in the control group, which may have attracted children whose parents are concerned about their eating habits and may have affected the result. Lastly, there was a small sample size in the CMPA group which reduced the ability to find significant differences. Further research should be done on a larger scale, with data collection in a more representative cross-section of Thailand's urban and rural areas.

## 5. Conclusion

Although this study found no significant difference in the prevalence of feeding difficulties between the CMPA group and the healthy control group, it is noteworthy that nearly half of all participants experienced feeding difficulties. The CMPA group also demonstrated a subtle difference in feeding behaviors, namely, slowness in eating. Notably, a decreased enjoyment of food and an increased desire to drink in CMPA children were found to be associated with more pronounced feeding difficulties. These findings suggest the potential impact of these factors on future feeding behaviors. It is important to emphasize that children diagnosed with CMPA exhibit reduced consumption levels of essential nutrients such as calcium, phosphorus, and zinc. As a result, it is advisable to actively promote adequate intake of these crucial nutrients among individuals with CMPA, accompanied by diligent monitoring of their nutritional status. This approach will ensure optimal growth and development in this population.

## Figures and Tables

**Table 1:** Baseline characteristics and anthropometric data of children in CMPA and healthy groups (*n* = 122).

	CMPA group (*n* = 30)	Healthy group (*n* = 92)	*p* value
Age (months)^1^	31.0 (14.0, 43.3)	40.0 (28.0, 53.8)	0.01
Gestational age (weeks)^2^	38.0 (38.0, 39.0)	38.0 (38.0, 40.0)	0.93
Birth weight (grams)^1^	3210.6 ± 364.9	3148.4 ± 350.9	0.41
Gender: boy, *n* (%)^3^	18 (60%)	53 (57.6%)	0.82
Duration of exclusive breastfeeding (months)^2^	6.0 (3.0, 6.0)	6.0 (4.0, 6.0)	0.86
Age at started complementary food (months)^2^	6.0 (6.0, 6.0)	6.0 (6.0, 6.0)	0.83
Main caregivers, *n* (%)^3^			
Parents	24 (80%)	77 (83.7%)	0.64
Others	5 (20%)	15 (16.3%)	
Main caregiver education, *n* (%)^3^			
Elementary school or less	5 (16.7%)	7 (7.6%)	0.24
Middle school	2 (6.7%)	6 (6.5%)	
High school	0 (0%)	10 (10.9%)	
Bachelor or more	23 (76.6%)	69 (75%)	
Family income (baht/month), *n* (%)^3^			
<30,000	3 (10%)	10 (10.9%)	0.50
30,000–49,999	8 (26.7%)	31 (33.7%)	
50,000–69,999	5 (16.7%)	22 (23.9%)	
≥70,000	14 (46.7%)	29 (31.5%)	
Weight for age *z*-score^1^	−0.24 ± 1.40	−0.12 ± 1.00	0.62
Height for age *z*-score^1^	−0.65 ± 1.37	−0.26 ± 1.06	0.11
BMI for age *z*-score^1^	0.24 ± 1.42	0.03 ± 1.32	0.46

^1^Continuous variables with normal distribution are presented as mean ± S.D, compared between two groups using an independent *t*-test. ^2^Continuous variables with nonnormal distribution are presented as median (IQR), compared between two groups using the Mann–Whitney *U* test. ^3^Categorical variables are presented as *n* (%), compared between two groups using The chi-squared test. Abbreviation: CMPA, cow's milk protein allergy; BMI, body mass index.

**Table 2:** Clinical characteristics of children in the CMPA group (*n* = 30).

Clinical characteristics	
Age at diagnosed CMPA (months)^1^	7.0 (3.8, 10.5)
Age at elimination of cow's milk (months)^1^	6.0 (3.0, 9.0)
IgE-mediated CMPA	27 (90%)
Number of food allergy, *n* (%)^2^	
1 (only cow's milk)	9 (30%)
2	5 (16.7%)
3	4 (13.3%)
4	1 (3.3%)
5	6 (20%)
6 or more	5 (16.7%)
Type of milk during feeding, *n* (%)^2^	
Breast milk	4 (13.3%)
eHF or AA	12 (40%)
Others, e.g., soy and mix (soy + eHF/AA)	14 (46.7%)
Allergic symptoms, *n* (%)^2^	
GI	15 (50%)
Non-GI	15 (50%)

^1^Continuous variables with nonnormal distribution are presented as median (IQR). ^2^Categorical variables are presented as *n* (%). Abbreviation: CMPA, cow's milk protein allergy; eHF, extensively hydrolyzed formula; AA, amino acid formula; GI, gastrointestinal.

**Table 3:** Dietary data of the children with CMPA and healthy group (*n* = 114)^1^.

	CMPA group	Healthy group	*p* value
Total calories (kcal/kg/day)^3^	86.86 (71.90, 99.37)	76.43 (59.52, 90.81)	0.13
Protein (g/kg/day)^3^	3.31 (2.66, 3.84)	3.11 (2.30, 3.65)	0.34
Calcium (mg/day)^3^	427.28 (196.02, 677.03)	598.31 (420.96, 811.34)	0.009
Phosphorus (mg/day)^2^	375.24 ± 185.86	667.73 ± 298.96	<0.001
Ferrous (mg/day)^3^	6.65 (4.58, 9.52)	4.63 (3.42, 6.68)	0.007
Zinc (mg/day)^3^	2.95 (2.20, 4.50)	3.76 (2.62, 5.45)	0.02

^1^Four participants in each group did not complete the data. ^2^Continuous variables with normal distribution are presented as mean ± S.D, compared between two groups using an independent *t*-test. ^3^Continuous variables with nonnormal distribution are presented as median (IQR), compared between two groups using the Mann–Whitney *U* test. Abbreviation: CMPA, cow's milk protein allergy.

**Table 4:** Feeding difficulties in children with CMPA compared to healthy children.

	CMPA group (*n* = 30)	Healthy group (*n* = 92)	*p* value
MCH-FS *T*-score^1^	58.00 ± 13.21	58.61 ± 11.05	0.80
MCH-FS *T*-score adjusted for age^1^	58.00 ± 1.75	58.61 ± 1.87	0.12
Classification of feeding difficulties^2^^,^^3^			
No feeding difficulties	19 (63.3%)	52 (56.5%)	0.70
Mild feeding difficulties	4 (13.3%)	21 (22.8%)	
Moderate feeding difficulties	2 (6.7%)	7 (7.6%)	
Severe feeding difficulties	5 (16.7%)	12 (13%)	

^1^Continuous variables with normal distribution are presented as mean ± S.D, compared between two groups using an independent *t*-test. ^2^Categorical variables are presented as *n* (%), compared between two groups using a chi-squared test. ^3^The degree of feeding difficulties was classified according to MCH-FS. Abbreviation: CMPA, cow's milk protein allergy; MCH-FS, Montreal Children's Hospital Feeding Scale; CEBQ, Child Eating Behavior Questionnaire.

**Table 5:** Factors associated with a higher feeding difficulties score in all participants (*n* = 122).

	Univariate linear regression		Multiple linear regression	
	*β*	*p* value	*β*	*p* value
Sex	-1.401	0.51		
Age	0.105	0.08		
Gestational age	-1.596	0.09		
Birth weight	0.003	0.88		
Duration of exclusive breastfeeding	-0.042	0.85		
Age at started complementary food	-0.447	0.76		
BMI for age *z*-score	-0.958	0.22		
CEBQ feeding behaviors				
Food responsiveness	-5.505	<0.001		
Emotional over-eating	-4.061	0.05		
Enjoyment of food	-10.769	<0.001	-7.724	<0.001
Desire to drink	2.571	0.002		
Satiety responsiveness	9.464	<0.001		
Slowness in eating	7.579	<0.001	2.537	0.002
Emotional under-eating	0.880	0.55		
Food fussiness	8.061	<0.001	3.062	<0.001

Abbreviation: CEBQ, Child Eating Behavior Questionnaire.

**Table 6:** Factors associated with a higher feeding difficulties score in cow's milk protein allergy group (*n* = 30).

	Univariate linear regression		Multiple linear regression	
	*β*	*p* value	*β*	*p* value
Sex	6.528	0.19		
Age	0.256	0.09		
Gestational age	-1.390	0.58		
Birth weight	-0.004	0.55		
Duration of exclusive breastfeeding	-0.417	0.50		
Age starting complementary food	2.596	0.72		
Age diagnosed CMPA	-0.144	0.75		
Age at elimination of cow's milk	0.088	0.87		
Number of food allergy	2.046	0.09		
Allergic symptoms	-1.600	0.75		
BMI for age *z*-score	-1.569	0.37		
CEBQ feeding behaviors				
Food responsiveness	-7.121	0.001		
Emotional over-eating	-10.268	0.03		
Enjoyment of food	-10.851	<0.001	-10.684	<0.001
Desire to drink	3.464	0.09	3.079	0.011
Satiety responsiveness	11.417	<0.001		
Slowness in eating	8.275	0.001		
Emotional under-eating	-4.471	0.202		
Food fussiness	8.358	0.001		

Abbreviation: CEBQ, Child Eating Behavior Questionnaire; CMPA, cow's milk protein allergy.

## Data Availability

The data that support the findings of this study are available from the initial author request.
